# Fatigue Acetabular Fracture after Lumbopelvic Instrumented Fusion in Elderly

**DOI:** 10.1155/2021/8962203

**Published:** 2021-09-29

**Authors:** Panagiotis Korovessis, Vasileios Tsekouras, Alkis Korovesis

**Affiliations:** ^1^Chief Orthopedic Department, General Hospital “Agios Andreas”, Tsertidoustr, 26224 Patras, Greece; ^2^General Hospital “Agios Andreas”, Tsertidoustr, 26224 Patras, Greece; ^3^Open University of Patras, 65-67 Haralabistr, 26224 Patras, Greece

## Abstract

**Purpose:**

Only several cases of acetabular “fatigue”/insufficiency fractures have been reported in elderly patients with osteoporosis. However, fatigue acetabular fracture below lumbopelvic fixation has not been published. This review reports on the frequency and mechanisms of acetabular fatigue fractures in elderly individuals, including postmenopausal osteoporosis, and presents a case of an acetabular “fatigue” fracture in association with lumbopelvic fusion.

**Methods:**

We report on a 71-year-old postmenopausal woman who underwent in our department a L2-pelvis instrumented fusion for failed lumbar decompression and interbody fusion performed in another institution. For at least one year, the patient was receiving antiosteoporotic treatment (Alendronate plus Calcium and Vitamin D) and was fully ambulatory without limping. Eighteen months following our surgery, the patient sought again our department because of increasing pain in her right hip and limping without trauma.

**Results:**

The physical examination disclosed painful passive motion in her right hip. The roentgenograms and CT-scans disclosed a transverse acetabular fracture with radiolucencies around both iliac screw tips, particularly the right. Additionally, a severe compression fracture of the 12^th^ thoracic vertebral body and upper endplate of the L2 vertebra was disclosed. We recommended open stabilization of the acetabulum and T12 and L2 vertebrae. Immediately before the planned surgeries, the patient had a serious heart infarct, and thus, surgeries were canceled by the patient's cardiologist because of the high perioperative risk. The patient and relatives denied further surgeries because of the heart disease. In the final telephone call and CT and roentgenographic evaluation that went to us after request, there was an acetabular pseudarthrosis in the right hip without however associated complaints. Since surgery was not accepted, the patient was prescribed Denosumab injection therapy plus Vitamin and Calcium supplement.

**Conclusion:**

This case report emphasizes the significance of follow-up observation of elderly patients with postmenopausal osteoporosis following lumbopelvic fusions, for possible fatigue acetabular and vertebral fractures. The authors speculate that this extremely rare acetabular “fatigue”/insufficiency fracture should be the result of increased repetitive mechanical forces acting around the acetabulum in association with osteoporosis.

## 1. Introduction

Pelvic insufficiency fractures typically occur in the sacrum and pubic rami and infrequently in the acetabulum in women with osteoporosis, rheumatoid arthritis, and abnormal spinal alignment [1–4]. In elderly patients, 50-83% of acetabular fractures are caused by a simple fall from a standing height onto the affected side [5, 6]. Rather than fracturing at the femoral neck or intertrochanteric region, the low-energy acetabular fractures occur in a typical pattern: impact of the greater trochanter onto the ground generates an anteromedial force transmission, driving the femoral head into the acetabular socket [7].

To our knowledge, acetabular fracture in association with spinopelvic construct has not yet been reported. We describe the first case of a unilateral “fatigue” transverse-type acetabular fracture that was observed 16 months following lumbopelvic revision surgery, associated with two lumbar vertebral body fractures without trauma, in an elderly woman with osteoporosis.

## 2. Case Report

**Figure 1:**
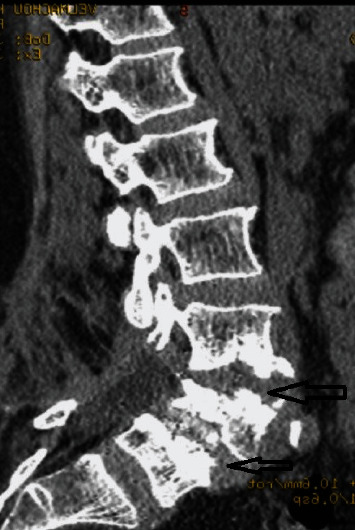
Preoperative CT-scan of a 71-year-old woman on admission.

**Figure 2:**
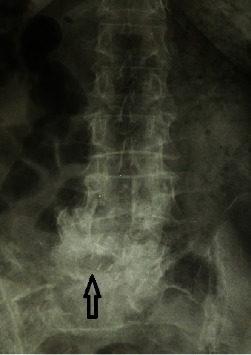
Preoperative anteroposterior roentgenogram of the patient. The arrow shows a previously inserted intervertebral PEEK cage.

A 71-year-old woman, with body mass index 26, sought our department because of increasing back pain. On admission, her hip motion was pain-free without limping or Trendelenburg. The active and passive motion in the lumbar spine was painful without neurologic findings in the lower extremities. In her previous history, the patient received wide laminectomy and interbody fusion with PEEK cage for spondylolisthesis and spinal stenosis, without additional pedicle screw fixation, in another institution two years ago (Figures 1 and 2).

**Figure 3:**
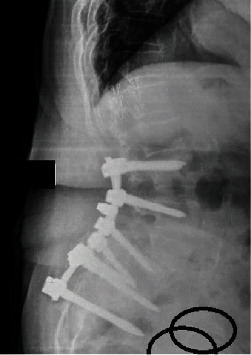
Preoperative lateral roentgenogram at one-year follow-up. L3 to lumbopelvic fixation.

**Figure 4:**
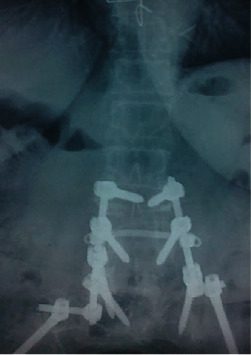
Postoperative anteroposterior roentgenogram of the lumbar spine.

She underwent in our department a spinopelvic fusion (L2 to pelvis) with iliac screws for lumbar pseudarthrosis L4-L5. In her previous history, the patient suffered from Sjogren's syndrome; osteoporosis (DEXA, *T*‐score = −3.1) under Alendronate, Calcium, and Vitamin D treatment; and unstable coronary heart disease. In the revision surgery, two iliac screws 85 and 75 mm long and 8 mm thick were inserted under fluoroscopy from the posterior superior iliac spine obliquely downwards to the ipsilateral acetabulum roof, respectively (Figures 3 and 4).

**Figure 5:**
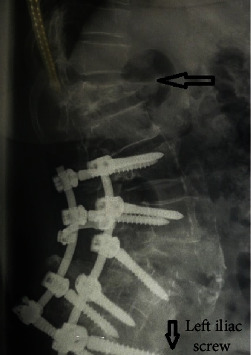
Lateral roentgenogram 18 months postoperatively showing a vertebral compression fracture of the L1 vertebra (arrow).

**Figure 6:**
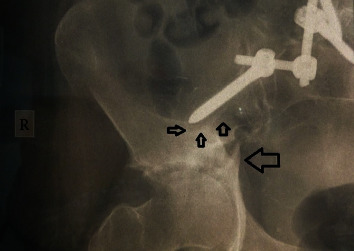
Anteroposterior roentgenogram of the right hemipelvis 18 months postoperatively showing a transverse-type Letournel fracture of the right acetabulum (big arrow). Small arrows show the radiolucent area around the tip of the right iliac screw. There is radiolucency around the iliac screw.

**Figure 7:**
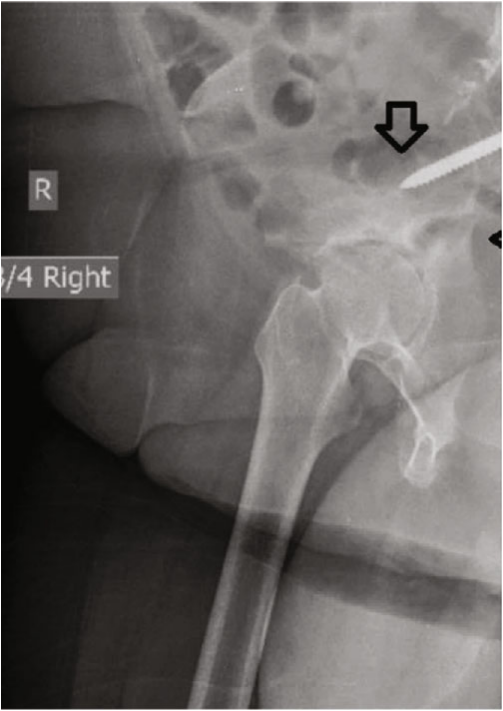
Right oblique Judet roentgenogram 18 months postoperatively showing the transverse fracture of the right acetabulum (long arrow) with disruption of the posterior acetabular column and the tip of the iliac screw (short arrow).

**Figure 8:**
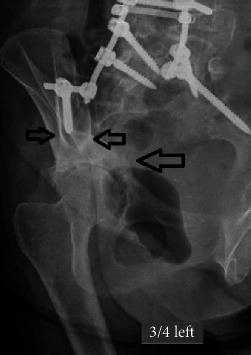
Oblique left Judet roentgenogram of the right hip 18 months postoperatively showing the transverse acetabular fracture. The left hemipelvis and particularly the acetabulum are intact. There is a radiolucent line around the tip of the left iliac screw. The right iliac screw has a central correct between the anterior and posterior iliac cortices.

The patient was discharged from the hospital one week postoperatively with a custom-made TLSO. She was followed consecutively and already at the postoperative evaluations; three and 12 months postoperatively, there was no limping or hip pain. Sixteen months following revision surgery, the patient sought us for increasing pain intensity in her right hip since 2 weeks without fall or other trauma. The patient was limping, and the passive movement in her right hip was painful. The roentgenographic examination disclosed a hemitransverse Letournel-type acetabular fracture in the right hip (Figures 5–8) and a severe compression fracture of the 12^th^ thoracic and 2nd lumbar-vertebra.

**Figure 9:**
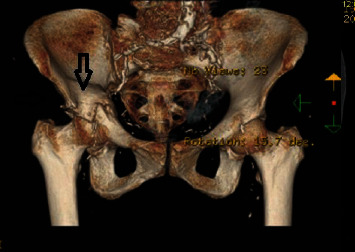
CT reconstruction image showing the transverse fracture line in the right hip. Note the proximity of the right iliac screw to the acetabular roof. Correct position of the iliac screws within the laminae of the pelvic bone bilaterally.

**Figure 10:**
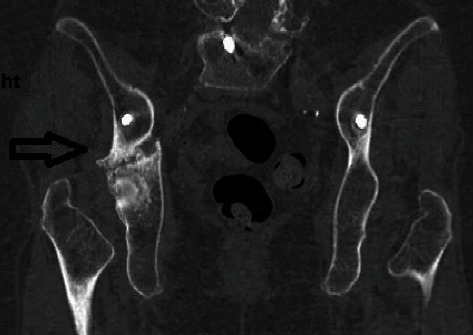
Frontal CT reconstruction image of the pelvis.

There was a continuous wide radiolucent line around the tip of the right iliac screw (Figures 7 and 8). We recommended ORIF for the acetabular fracture and stabilization of the L1-vertebral fracture. The pelvis CT-scan showed a pseudarthrosis in the right acetabulum close to the right iliac screw tip (Figures 9 and 10).

At that time, the planned surgeries had to be canceled because the patient had another heart infarct one week before the planned acetabular ORIF surgery.

**Figure 11:**
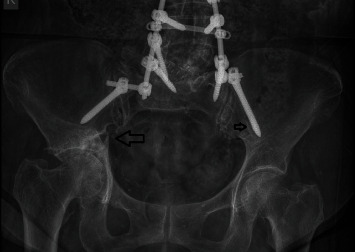
Anteroposterior pelvis roentgenogram showing the acetabular fracture (right, thick arrow) and radiolucent line around the left iliac screw (small arrow).

**Figure 12:**
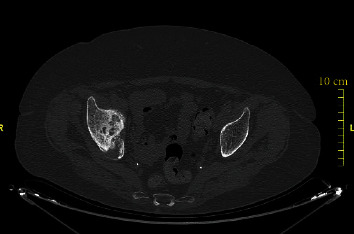
Axial CT-scan view showing the acetabular nonunion on the right.

**Figure 13:**
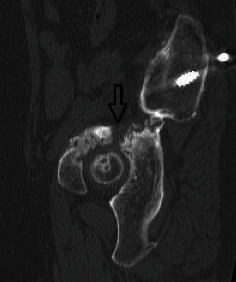
Sagittal right pelvis reconstruction CT-image showing the acetabular pseudarthrosis line (arrow).

During the last telephonic call, one month before the writing of this revised paper, the patient reported no pain in the hip and spine. The relatives made in her local radiologist new AP plain pelvis roentgenogram (Figure 11) that showed atrophic pseudarthrosis and radiolucencies around the iliac screws and CT-scans (Figures 12 and 13), and the patients' relatives sent it to us. There was now an evident atrophic pseudarthrosis in the site of the transverse acetabular fracture (Figures 12 and 13, arrow). Following that and with the history of heart infarct, the patient and her relatives denied further surgeries. Since surgery was not possible at this time, Denosumab injection therapy plus Vitamin and Calcium supplement was applied to this fragile patient.

## 3. Discussion

Contemporary spinopelvic fixation and fusion with iliac screws is one of the most biomechanically sound methods of stabilizing multisegment constructs usually made for adult degenerative lumbar spine disease. It was shown that the incorporation of the ilium into the construct decreases the rate of failure and enhances spinal fusion comparing with lumbosacral fixation [5, 6]. The “fatigue” acetabular fracture that we present here might be the first ever described. This acetabular fracture might be of a different etiology than the “insufficiency” acetabular fracture types [2, 3].

There are some similarities between insufficiency acetabular fracture in the elderly and this “fatigue” acetabular fracture in an elderly patient with significant osteoporosis who received an instrumented lumbopelvic fusion. A typical insufficiency acetabular fracture is difficult to recognize in plain roentgenograms when it appears as a break in the acetabular cortex or asymmetric linear arc of sclerosis parallel to the acetabular roof [2, 3], while it is easily recognized when it appears as anterior column/anterior wall Letournel type and anterior column-posterior hemitransverse pattern type [5–11]. Insufficiency acetabular fracture takes several weeks to be diagnosed. Additional spine and/or pelvic fractures may help to identify a lesion in the acetabulum as an “insufficiency” fracture as in our case. Risk factors for acetabular insufficiency fractures are postmenopausal osteoporosis, rheumatoid arthritis, corticosteroid therapy, and spinal alignment abnormalities [2, 4, 12–14].

We speculate that the mechanism of the unilateral acetabular fracture in our patient might be as follows: The iliac screws transfer the loads from the spine down to the hips through the supra-acetabular region. The simultaneous presentation of acetabular fracture and lumbar compression fracture above the fusion shows that increased stresses were applied below the iliac screws and above the acetabulum. The transverse fracture of the right acetabulum occurred few millimeters below the iliac tip that was covered with radiolucency. The CT-scan showed the proximity of the iliac screw to the acetabular pseudarthrosis. The patient denied the proposed reconstruction surgery because of severe infarct.

Spine surgeons should be aware of acetabular fatigue fracture in the elderly after lumbopelvic fixation with iliac screws in patients with significant osteoporosis.

## Data Availability

All data for this patient are in the Clinic Archives.

## References

[1] Pentecost R. L., Murray R. A., Brindley H. H. (1964). Fatigue, insufficiency, and pathologic fractures. *JAMA*.

[2] Cooper K. L., Beabout J. W., McLeod R. A. (1985). Supraacetabular insufficiency fractures. *Radiology*.

[3] Davies A. M., Bradley S. A. (1991). Iliac insufficiency fractures. *The British Journal of Radiology*.

[4] Peh W. C. G., Gough A. K. S., Sheeran T., Evans N. S., Emery P. (1993). Pelvic insufficiency fractures in rheumatoid arthritis. *British Journal of Rheumatology*.

[5] Ferguson T. A., Patel R., Bhandari M., Matta J. M. (2010). Fractures of the acetabulum in patients aged 60 years and older. *Journal of Bone and Joint Surgery (British)*.

[6] Vanderschot P. (2007). Treatment options of pelvic and acetabular fractures in patients with osteoporotic bone. *Injury*.

[7] Odate S., Shikata J., Kimura H., Soeda T. (2013). Sacral fracture after instrumented lumbosacral Fusion. *Spine*.

[8] Zelle B. A., Cole P. A. (2011). Open reduction and internal fixation of complex geriatric acetabular fracture. *Operative Techniques in Orthopaedics*.

[9] Lebwohl N. H., Cunningham B. W., Dmitriev A. (2002). Biomechanical comparison of lumbosacral fixation techniques in a calf spine model. *Spine*.

[10] Kuklo T. R., Bridwell K. H., Lewis S. J. (2001). Minimum 2-year analysis of sacropelvic fixation and L5-S1 fusion using S1 and iliac screws. *Spine*.

[11] Culemann U., Holstein J. H., Köhler D. (2010). Different stabilisation techniques for typical acetabular fractures in the elderly--A biomechanical assessment. *Injury*.

[12] Jeffcoat D. M., Carroll E. A., Huber F. G. (2012). Operative treatment of acetabular fractures in an older population through a limited ilioinguinal approach. *Journal of Orthopaedic Trauma*.

[13] Scemama C., D’astorg H., Guigui P. (2016). Sacral stress fracture after lumbar and lumbosacral fusion. How to manage it? A proposition based on three cases and literature review. *Orthopaedics& Traumatology: Surgery & Research*.

[14] Mathews V., McCance S. E., O’Leary P. F. (2001). Early fracture of the sacrum or Pelvis. *Spine*.

